# Oral hygiene practices and utilization of oral healthcare services among in-school adolescents in Calabar, Cross River State, Nigeria

**DOI:** 10.11604/pamj.2020.36.300.25102

**Published:** 2020-08-18

**Authors:** Divine-Favour Chichenim Ofili, Ekpereonne Babatunde Esu, Regina Idu Ejemot-Nwadiaro

**Affiliations:** 1 Department of Epidemiology, Biostatistics and Occupational Health, McGill University, Montreal, Canada,; 2 Department of Public Health, College of Medical Sciences, University of Calabar, Calabar, Nigeria

**Keywords:** Oral healthcare services, utilization, awareness, oral hygiene practices

## Abstract

**Introduction:**

oral health mirrors an individual´s general health, hence, proper care should be taken to prevent oral diseases and conditions. An estimated 3.9 billion people worldwide are affected by oral conditions, and adolescents are at increased risk due to diet choices and hormonal changes. This study aimed at determining the oral hygiene practices, awareness and utilization of oral healthcare services among in-school adolescents in Calabar Municipality.

**Methods:**

a descriptive cross-sectional study design was employed in studying these variables among in-school adolescents (10-19 years) in secondary schools (JSS1-SSS2) using a self-administered, semi-structured questionnaire. Data were entered and analyzed with EPI-Info. Cross tabulations of variables were conducted using Chi-square test with significance level of 5%.

**Results:**

a total of 335 students participated in the study with 228 (68.1%) and 136 (40.6%) reporting that they are aware of and have utilized oral healthcare services (OHS), respectively. There was no statistically significant association between age (p=0.923), gender (p=0.351) and type of school (p=0.497) respectively with awareness and utilization of OHS. Toothache/pain and presumed good dental health respectively were the main reasons for the usage and non-utilization of these services. Time-to-use of oral health services after the onset of toothache/pain was within five days (75.7%).

**Conclusion:**

the utilization rate of OHS did not match up to the level of awareness of these services with pain being the main driver for utilization. Increased awareness of oral healthcare through health education and oral healthcare demonstrations among adolescents is highly recommended as part of school health programs.

## Introduction

Oral health is an integral component of our overall wellbeing because the oral cavity is the doorway to the body. Oral health becomes all the more necessary because of the aesthetic nature and social value it commands. Further, the care given to this part of the body may prevent several diseases. Oral health as defined by the World Health Organization (WHO) [1] is “a state of being free from mouth and facial pain, oral and throat cancer, oral infection and sores, periodontal (gum) disease, tooth decay, tooth loss and other disorders that limit an individual´s capacity in biting, chewing, smiling, speaking and psychosocial wellbeing”. It is evident from this definition that oral health is an aspect of health affecting the general health and wellbeing of an individual. The limitation to biting and chewing means the individual to an extent will be unable to eat food rich in nutrients needed for the body´s development. Man as a social being should be able to interact freely with others, but with oral disorders that affect the individual´s psychosocial well-being, his ability to smile, speak and at times love is hugely hampered. An estimated 3.9 billion people worldwide are affected by oral conditions [2]. Due to this high prevalence rate, the WHO [1] added oral health to the list of prioritized non-communicable diseases (NCDs), especially as it shares common risk factors with other NCDs such as diabetes, cardiovascular diseases and cancer. Diseases such as diabetes and HIV/AIDS affect the oral health of an individual, but poor oral health on its own can aggravate these health conditions [3].

In Nigeria, based on a previously conducted national oral health survey, the prevalence rates of dental caries and periodontal diseases stand at 30% and >80% [4]. The burden in Nigeria shows a pattern of deterioration which is closely linked to poverty and the poor economic growth in the country. These figures may be a gross underestimation of the oral condition burden in Nigeria since the rural areas rarely have functional oral health facilities, with poor accessibility to the facilities and services. Also, the populace that patronizes unorthodox healthcare providers in the country is not considered. The occurrence of oral diseases is higher among poor and disadvantaged population groups in both developing and developed countries of the world, with the most commonly observed conditions being dental caries and periodontal diseases. Others include; cleft lips and palate, oral cancer, noma and oro-dental trauma [1, 5-8]. The most common risk factors of oral diseases are poverty, ignorance/low level of awareness, poor oral hygiene, as well as unhealthy lifestyles and behaviours such as tobacco use and excessive alcohol consumption [8, 9]. Dental caries or cavities, simply known as tooth decay, is prevalent amongst children and is said to be the world´s most widespread chronic disease. Dental caries affects about 60-90% of students globally [1]. In Nigeria, it affects about 6-23% of the general public, with about 90% of the cases left untreated [7]. Poverty increases the vulnerability of individuals to dental caries; this means that about 80% of people living in sub-Saharan Africa are at increased risk [9]. Periodontal disease, otherwise known as gum disease which is most likely to result in tooth loss, is common among middle-aged adults and affects about 15-20% of this population [1]. The prevalence rate of gum diseases with deep pocketing among Nigerians 15 years and above, as reported by Akpata [6] was 15-58%. This condition is spreading more rapidly in undernourished populations [10].

Though Nigeria has a low prevalence of oral health diseases compared to some western countries, the problem is that majority of the affected go untreated [7]. This may be attributed to lack of awareness, unaffordability of dental care services, and the grossly inadequate number of dental care professionals to meet the enormous dental care needs of the population [6, 8, 11]. These factors may result in patients resorting to unskilled personnel for their oral health needs and reporting to a professional only when the problem has worsened. Even more, not much attention seems to be given to oral health promotion in Nigeria as a whole and Cross River State in particular. School children, youths and the elderly are important target groups when talking about oral health, with adolescents being the highest risk group for dental caries [1, 12]. Adolescents are said to be between ages 10-19 years and are characterized by eating refined foods (with high sugar content), with body image and whole outlook concerns. It is also at this stage that relationships are formed and so building their social image is important to them. This population forms a significant part of Nigeria´s entire population (33%), hence, attention should be given to their oral and general health needs to improve their health now and when they are older [13]. Oral healthcare needs are highly underscored in adolescents, not only because of the high prevalence of oral disease conditions in this age group but for aesthetics, body image and social values attached to the oral cavity. Many people in this age group are still highly dependent on their caregivers, and this may influence their utilization of oral health services, as the adolescent(s) may be willing to use the services, but their caregivers may not see the need. Thus, improving oral health in this population will be very critical not only in promoting good health but also in improving socio-psychological relationships. This paper therefore aimed to determine the oral hygiene practices, awareness and utilization of oral healthcare services (OHS) by in-school adolescents in Calabar Municipality.

## Methods

A descriptive cross-sectional study design was adopted for this study carried out on in-school adolescents between the ages of 10-19 years in Calabar Municipality Local Government Area, Cross River State. The study tool was a self-administered, semi-structured questionnaire which obtained information on respondents´ socio-demographic data, oral hygiene practices, awareness and utilization of OHS and factors that influence the use of OHS. A sample size of 350 was determined to be adequate assuming the utilization rate of 27.9% for oral health services among in-school adolescents [13], 95% confidence level, 5% margin of error and 10% adjustment for non-response. Simple random sampling (lottery method without replacement) was used to select ten secondary schools (5 each from public and private schools registered in the municipality). A total of 35 students (7 each from 5 class levels) in Junior and Senior secondary school were selected from each of the ten schools for this study.

Data entry and analysis was conducted using EPI-Info (Version 7). Results were expressed using descriptive statistics. Associations between variables were tested using Chi-square statistical test and significance level was set at 5%. Ethical clearance to conduct this study was obtained from the Ethics Committee of the Department of Public Health, University of Calabar, Calabar. Permission to access schools and students was sought from the Cross River State Ministry of Education and through the Parent-Teachers Association of selected schools. Also, assent for participation was sought from the adolescents. Participation in the study was voluntary, and respondents were free to withdraw their participation from the study. All data were treated with anonymity and confidentiality.

**Inclusion criteria:** in-school adolescents in secondary schools in Calabar Municipality aged between 10 and 19 years and gave verbal consent to participate in the study.

**Exclusion criteria:** out-of-school adolescents, individuals not in secondary school and those that don´t fall within the age group needed.

## Results

**Socio-demographic characteristics of respondents:** a total of 350 copies of the self-administered questionnaire were distributed to in-school adolescents in Calabar Municipality, Cross River State. Only 335 copies of the questionnaire were completely and correctly filled (response rate of 98.2%) and included in the analysis. The mean age among the in-school adolescents was 13.4±1.85 years with respondents aged between 10 and 19 years. More respondents were female, 189 (56.4%) and 171 (51%) of the respondents attended private schools.

**Awareness of oral healthcare services and oral hygiene practices of respondents:** overall, 68.1% respondents were aware of OHS, but a lower percentage of the respondents (41.8%) knew where to access OHS. Most of the respondents, 318 (94.9%), identified the dentist/hospital as an oral healthcare service provider (Table 1). The results presented in Table 1 show that approximately two-third of the respondents (66.9%) brushed their teeth twice a day while 17% and 15.2% brush once and thrice everyday respectively. Majority of the respondents, 98.8%, use toothbrush and toothpaste to brush their teeth, others make use of only toothbrush, soap and brush and chewing sticks. However, about half of the respondents, 54.3%, had never used a dental floss (Table 1).

**Table 1:** awareness of oral healthcare services and oral hygiene practices

Variables	Frequency	Percentage (%)
**Aware of OHS**		
Yes	228	68.1
No	107	31.9
Total	335	100
**Knew where to access OHS**		
Yes	140	41.8
No	195	58.2
Total	335	100
**Place where OHS can be accessed**		
Dentist/hospital	318	94.9
Patent medicine vendors	4	1.2
Patent medicine shop	1	0.3
Traditional healer	5	1.5
Did not know	7	2.1
Total	335	100
**Frequency of teeth brushing**		
Once a day	57	17.0
Twice a day	224	66.9
Thrice a day	51	15.2
Irregularly a week	3	0.9
Total	335	100
**Materials used for oral hygiene**		
Toothbrush and toothpaste	331	98.8
Toothbrush only	2	0.6
Chewing stick	1	0.3
Soap and brush	1	0.3
Total	335	100
**Utilization of dental floss**		
Once a day	52	15.5
Twice a day	48	14.3
After each meal/ after eating	53	15.8
Never	182	54.3
Total	335	100

**Figure 1:**
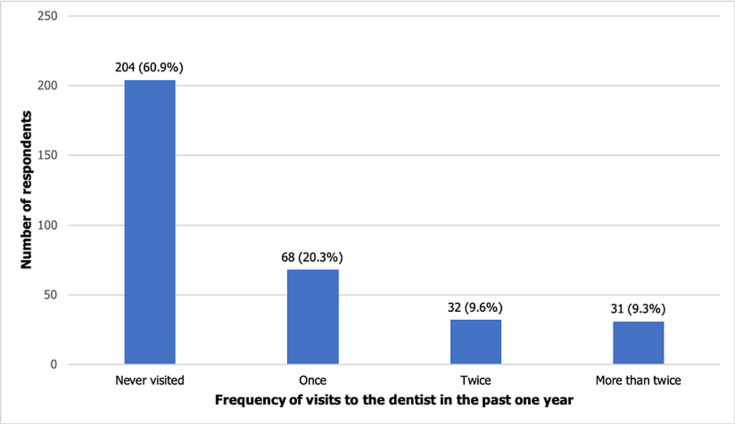
utilization of oral healthcare services in the past one year

**Utilization of oral healthcare services:** only 40.6% respondents reported the use of an oral healthcare service. Of these, 19.9% utilized the service due to toothache and 78 (57.4%) to prevent tooth decay. 127 (93.4%) in-school adolescents who had used an oral healthcare service received the service from a dentist/hospital (Table 2). Of the 236 respondents who have suffered a toothache before, 176 (74.6%) sought help to stop the pain and 81.8% of this group sought help from a dentist/hospital. Help from a dentist or hospital was sought within 5 days after onset of toothache by 75.7% of the respondents (Table 2). Presumed good dental health (22.7%), fear and dental anxiety (21.9%), cost (9.8%) and inability to locate a dental clinic (13.7%) were identified by the respondents as some of the factors influencing the use of OHS (Table 2). In the last year, 60.9% of the respondents never visited the dentist, with the rest visiting at least once (Figure 1).

**Table 2:** utilization of oral healthcare services

Variables	Frequency	Percentage (%)
**Ever used oral healthcare services**		
Yes	136	40.6
No	182	54.3
Did not know	17	5.1
Total	335	100
**Reason for use**		
To prevent tooth decay	78	57.4
Toothache	27	19.9
For tooth extraction	6	4.4
Routine dental check-up	23	16.9
Did not know	2	1.4
Total	136	100
**Place where OHS accessed**		
Traditional Healer	3	2.2
Dentist/hospital	127	93.4
Patent medicine vendors	4	2.9
Free medical outreach	1	0.7
Did not know	1	0.7
Total	136	100
**Ever had tooth pain/ache**		
Yes	236	70.4
No	99	29.6
Total	335	100
**Sought help for toothache**		
Yes	176	74.6
No	60	25.4
Total	236	100
**Care seeking for toothache**		
Traditional healer	3	1.7
Patent medicine shop	23	13.1
Patent medicine vendors	6	3.4
Dentist/hospital	144	81.8
Total	176	100
**Time to hospital visit after the ache started**		
0-5 days	109	75.7
6-20 days	23	16.0
20 days- 1 month	5	3.5
1-5 months	4	2.8
Above 5 months	3	2.1
Total	144	100
Factors affecting utilization*		
Cannot locate a dental clinic	113	13.7
Cost of utilization	81	9.8
Use herbal drugs/ treatment (self-treatment)	37	4.5
Scared of the equipment and tools used	92	11.1
No reason to go there	88	10.6
Not allowed to go by parents	49	5.9
Long waiting time	45	5.4
Teeth is in condition	188	22.7
Unfriendly hospital/ clinic staff	27	3.3
Scared of losing my tooth/teeth	89	10.8
Use unskilled health workers	18	2.2

**Association between socio-demographic variables and awareness of oral healthcare services:** we found that more students from public schools and females are aware of and have utilized OHS, although no strong associations were observed (p=0.497 and 0.351 respectively, Table 3).

**Table 3:** association between socio-demographic characteristics of the respondents and awareness of oral healthcare services

Demographic variables	Ever utilized an oral healthcare service			
**Yes n (%)**	**No n (%)**	**Total n (%)**	**P- value**
**Type of school**				0.497
Private	57 (53.3)	50 (46.7)	107 (100)	
Public	59 (48.8)	62 (51.2)	121 (100)	
**Gender**				0.351
Female	60 (49.2)	62 (50.8)	122 (100)	
Male	56 (52.8)	45 (47.2)	106 (100)	
**Age (in years)**				
10-12	36	36	72	0.923
13-15	66	61	127	
16-19	14	15	29	

n= 228 (only those aware of oral healthcare services)

## Discussion

A service is considered optimum when access to and use of such a service is at least 90%, however, utilization of OHS from this study is barely 40%. Although our findings are closely related, the utilization rate recorded from this study (40.6%) is higher than the rates from earlier studies [9, 14]. The reasons for usage ranged from having toothache to routine dental check-up. Presumably, a good number of respondents used the service to prevent a possible tooth decay due to the toothache/pain they experienced. In line with the findings of other studies, 43% of the respondents who sought for help from an oral healthcare service provider/clinic did so due to a toothache. This is consistent with the conclusion of researchers that pain is the main reason for seeking dental care/utilizing an oral healthcare service [9, 15]. This may be because pain poses as an appealing factor for seeking care, as it may be signifying a more complex problem [14]. Regular visits to dental clinics is not a tradition adopted by Nigerians as 60.9% of the total respondents and 37.5% of the respondents who reported to have ever utilized OHS never visited/accessed such services in the past year. Possibly because they do not suffer from any oral conditions and feel complacent with their oral health. It therefore validates the conclusion that OHS are utilized for mainly curative and not preventative purposes, and that routine dental check-up is not a well-practiced culture in Nigeria and developing countries as a whole [16]. In a more positive response to pain, most of the respondents who reported experiencing toothache sought for help from a dentist within 5 days of pain onset. This suggests that this population and their caregivers do not wait for the condition to worsen before seeking for help. It is presumed that the rate of awareness of oral health and OHS is usually higher than the rate of utilization of these services. Perhaps so because people have to be aware of the service before they utilize it [7]. A previous study [17] reported that most of their respondents had good knowledge about routine dental check-up which did not translate into good practice.

This opinion is reflected in our results as just 34.6% of the respondents who were aware of these services had used them. In the same vein, another study opined that low awareness results in low utilization of OHS [18]. We found that more students in public schools were aware and had utilized OHS than students enrolled into private schools, although the difference was not statistically significant. This is in contrast to the belief held by most people that children enrolled in private schools show better preventive practices including regular visits to the dentist than those enrolled in public schools [17]. Based on this study, one cannot determine the exact cause of this variance. Compared to their male counterparts, more female reported knowing and using these services. This resonates with a previous study which found that a significantly higher proportion of females practice oral healthcare [18]. It may be because females tend to be more conscious of their outward appearance and beauty than the males, highlighting gender as a critical factor in oral care among adolescents. It was also discovered from the study that the younger adolescents (10-14 years) were aware and utilized OHS (75%) more than the older adolescents (15-19 years, 25%). This is in dissonance with earlier findings that the older adolescents had visited a dentist more than the younger adolescents in the last year preceding the study [19]. Age is considered a critical factor in the utilization of OHS, however, one study which also focused on in-school adolescents concluded that it cannot predict the utilization of such services [14]. This study went further to explain that more junior students tend to use OHS because of the “low pain threshold” in younger students. A possible explanation to our finding that the JSS 2 class had the most respondents who have utilized and are aware of OHS. Additionally, those within this class may have other influences for the use of OHS other than school. Class has been previously reported as a predictor of periodontal diseases as it had significant impact on the periodontal status of the students [8].

The main reason for non-utilization was presumed good oral health, possibly due to the lack of a motivating factor such as pain or an oral condition [14]. The second commonest reason was the respondents´ lack of knowledge on where to find a dental clinic, and this is resultant from the inadequacy of oral healthcare centres to cater for the oral health needs of the population. Dental anxiety and fear, including the fear of dental equipment and fear of losing teeth were also factors influencing the use of OHS. This fear may be a perceived or accumulated fear, as people presumably believe that a visit to the dentist results in tooth extraction. This highlights low awareness of OHS, as many people do not know that preventive services are offered in dental clinics and its advantages. Adolescents and the general public should therefore be encouraged to take up OHS for preventative reasons, pointing out that these visits would help prevent tooth loss. A good number of respondents also pointed out that they/their parents found it expensive. Although the cost of OHS is lower than that of most developed countries, it is however still considered expensive by a significant proportion of the population in developing countries [4]. It is worthy of note that 80% of people living in sub-Saharan Africa are considered to be living below poverty level [9]. Further, a great proportion of this group are dependent on others to meet their health needs and if their parents/guardians are unable to provide/cover these needs or don´t see a need to take their children/wards to see a dentist, such adolescents may be unable to utilize OHS [14, 20].

**Limitations of the study:** using a comprehensive questionnaire, we were able to determine the oral hygiene practices and capture the factors influencing the use of OHS by in-school adolescents, however, we had some limitations. Data was only obtained from in-school adolescents, limiting the generalisation of findings of this study as out-of-school adolescents oral health needs were not covered. As well, secondary data sources on oral health utilisation in health facilities in the study area which could have provided trends were not used.

## Conclusion

This study revealed that most in-school adolescents were aware and had high knowledge of where to access OHS. Although the utilization rate was relatively low, the study population responded promptly to pain by visiting the dentist. The main reason for utilization was pain, while the main reason for non-utilization was perception of optimal dental health (indicating utilization of OHS for mostly symptomatic reasons) and cost of treatment. It is important to note that most established oral diseases and conditions are irremediable, will last a lifetime and influence the quality of life and general health. This heightens the need to increase the awareness and consequently, utilization of OHS for both preventative and curative purposes (with focus on the preventive aspect) to prevent conditions and complications related to oral health in the nearest future. We therefore recommend that the cost of oral health services should be reduced by the government to increase utilization. This can be done by including oral health in health insurance schemes. Although the ‘basic package of oral care‘ within the PHC framework is one of the strategies for oral health promotion by the Federal Ministry of Health [21], it is yet to be implemented. Implementation of this section of the policy will improve access to OHS, increase utilization of such services and contribute to the achievement of Universal Health Coverage. The mouth is the mirror of the whole body, therefore knowledge on how to take care of it is of most importance.

### What is known about this topic

- Previous studies conducted on adolescents in different parts of Nigeria show that knowledge of oral hygiene practices and healthcare services do not translate to use of these services;
- Pain or an existing oral condition is the main motivation for use of oral care services among this population.

### What this study adds

- Our study confirms the mismatch between knowledge and use of OHS by adolescents but shows an improvement in time-to-response to toothache/pain by the study population;
- The need to improve awareness and promote the preventive use of OHS (not just curative purposes) amongst the adolescents is underscored in this study. Imbuing this behavior earlier in life results in less oral health complications in future;
- This study adds to the body of evidence as no previous research assessing the awareness and utilization of OHS by adolescents in Calabar, Nigeria has been conducted.
